# Supplementary material for: Batch effects removal for microbiome data via conditional quantile regression
Source: Nat Commun. 2022 Sep 15;13:5418. doi: 10.1038/s41467-022-33071-9 (PMC9477887; doi:10.1038/s41467-022-33071-9)
Supplement: Supplementary file 1 — Supplementary Information [file 41467_2022_33071_MOESM1_ESM.pdf]

Supplementary information of "Batch effects removal for microbiome data via conditional quantile regression"

Wodan Ling, Jiuyao Lu, Ni Zhao*, Anju Lulla, Anna M. Plantinga, Weijia Fu, Angela Zhang, Hongjiao Liu, Hoseung Song, Zhigang Li, Jun Chen, Timothy W. Randolph, Wei Li A. Koay, James R. White, Lenore J. Launer, Anthony A. Fodor, Katie A. Meyer, and Michael C. Wu*

Additional details of methods

Conditional quantiles of microbial counts Y_i.

By Model (2), we model a fine sequence of conditional quantiles of $W_i|Y_i > 0$. Due to the one-to-one relationship between quantiles of $W_i|Y_i > 0$ and quantiles of $Y_i|Y_i > 0$[1], together with Model (1), we have the entire conditional quantile function of Y_i piecewise defined,

$$\mathrm{Q}_{Y_i}(\tau|\mathbf{X}_\mathrm{i}) = \mathrm{I}\{\tau > 1 - \pi(\boldsymbol{\theta}^\mathrm{L}, \mathbf{X}_\mathrm{i})\} \cdot \lceil \mathbf{X}_\mathrm{i}^\mathrm{T}\boldsymbol{\theta}^\mathrm{Q} \circ \Gamma(\tau; \mathbf{X}_\mathrm{i}, \boldsymbol{\theta}^\mathrm{L}) - 1 \rceil, \qquad (\text{Supp. }1)$$

where $\pi(\boldsymbol{\theta}^\mathrm{L}, \mathbf{X}_\mathrm{i}) = \mathrm{P}(Y_i > 0|\mathbf{X}_\mathrm{i})$ and $\Gamma(\tau; \mathbf{X}_\mathrm{i}, \boldsymbol{\theta}^\mathrm{L}): (1 - \pi(\boldsymbol{\theta}^\mathrm{L}, \mathbf{X}_\mathrm{i}), 1) \to (0,1)$ is a one-to-one mapping from the target quantile level τ of Y_i to the nominal quantile level τ_s of $W_i|Y_i > 0$ in Model (2). Specifically, for a τ below the change point $\pi(\boldsymbol{\theta}^\mathrm{L}, \mathbf{X}_\mathrm{i})$, the outcome Y_i falls in the "absence range", thus its corresponding quantile is zero. For a τ beyond the change point, $\Gamma(\tau; \mathbf{X}_\mathrm{i}, \boldsymbol{\theta}^\mathrm{L})$ tells which quantile of $W_i|Y_i > 0$ determine the value of Y_i at τ. Mathematically,

$$\boldsymbol{\theta}^\mathrm{Q} \circ \Gamma(\tau; \mathbf{X}_\mathrm{i}, \boldsymbol{\theta}^\mathrm{L}) = \boldsymbol{\theta}^\mathrm{Q}(\tau_s), \tau_s = \Gamma(\tau; \mathbf{X}_\mathrm{i}, \boldsymbol{\theta}^\mathrm{L}) = \frac{\tau - \{1 - \pi(\boldsymbol{\theta}^\mathrm{L}, \mathbf{X}_\mathrm{i})\}}{\pi(\boldsymbol{\theta}^\mathrm{L}, \mathbf{X}_\mathrm{i})},$$

and the mapping is derived by

$$\begin{aligned}\tau &= \mathrm{P}\{Y_i \le \mathrm{Q}_{Y_i}(\tau|\mathbf{X}_\mathrm{i})|\mathbf{X}_\mathrm{i}\} \\ &= \{1 - \pi(\boldsymbol{\theta}^\mathrm{L}, \mathbf{X}_\mathrm{i})\} + \pi(\boldsymbol{\theta}^\mathrm{L}, \mathbf{X}_\mathrm{i})\mathrm{P}\{W_i \le \lceil \mathrm{Q}_{W_i}(\tau_s|\mathbf{X}_\mathrm{i}, Y_i > 0) - 1 \rceil + U|\mathbf{X}_\mathrm{i}, Y_i > 0\}.\end{aligned}$$

Apart from the characteristics mentioned in the manuscript, another merit of the two-part quantile regression model is that it allows nonlinear associations between quantiles of Y_i and the covariates $\mathbf{X}_\mathrm{i}$, though the logit of probability being present, and every quantile of the non-zero part are modelled by linear models. To give a concrete example, suppose that for one subject, the true likelihood of the investigated taxon being present in his gut (from Model

(1)) is $\pi = 0.8$. Based on the mapping function above, the conditional median abundance of this taxon is determined by the $\tau_s = \frac{0.5-(1-0.8)}{0.8} = 0.38$th quantile of W_i. If we change his assignment from placebo to treatment, how would the conditional median of the taxon abundance change accordingly? Suppose the true probability of having the taxon increases to $\pi = 0.9$ after he receives treatment. The conditional median would then be determined by the $\tau_s = \frac{0.5-(1-0.9)}{0.9} = 0.44$th quantile of W_i. The difference in conditional median is thus nonlinear, which is a composite effect from the two parts.

Piecewise estimation strategy.

Parameters of the two-part model (1)(2) can be readily estimated by regressing $\mathrm{I}(Y_i > 0)$ on $\mathbf{X}_\mathrm{i}$ using logistic regression and regressing the non-zero Y_i on the corresponding $\mathbf{X}_\mathrm{i}$ using linear quantile regression. As the variance of quantile regression estimate is inverse proportional to the local density, i.e., $\mathrm{var}\{\widehat{\boldsymbol{\theta}}^{\mathrm{Q}}(\tau)\} \to \infty$ as $\tau \to 0$, the quantile estimate is not stable at the change point $1 - \pi(\boldsymbol{\theta}^{\mathrm{L}}, \mathbf{X}_\mathrm{i})$, and might blow up to a value with extraordinarily large magnitude. To achieve a reliable estimation of the conditional quantile function $\hat{Q}$, we use a piecewise strategy:

1. Estimate the probability of presence, $\pi(\widehat{\boldsymbol{\theta}}^{\mathrm{L}}, \mathbf{X}_\mathrm{i}) = \exp(\mathbf{X}_\mathrm{i}^{\mathrm{T}}\widehat{\boldsymbol{\theta}}^{\mathrm{L}})/\{1 + \exp(\mathbf{X}_\mathrm{i}^{\mathrm{T}}\widehat{\boldsymbol{\theta}}^{\mathrm{L}})\}$
2. Select a constant $\delta \in (0, \frac{1}{2})$, divide the support of the target quantile levels $(0,1)$ of Y_i into three sub-intervals:

$$A_n = \{\tau: 0 < \tau < 1 - \pi(\widehat{\boldsymbol{\theta}}^{\mathrm{L}}, \mathbf{X}_\mathrm{i})\},$$
$$B_n = \{\tau: 1 - \pi(\widehat{\boldsymbol{\theta}}^{\mathrm{L}}, \mathbf{X}_\mathrm{i}) \le \tau \le 1 - \pi(\widehat{\boldsymbol{\theta}}^{\mathrm{L}}, \mathbf{X}_\mathrm{i}) + n^{-\delta}\},$$
$$C_n = \{\tau: 1 - \pi(\widehat{\boldsymbol{\theta}}^{\mathrm{L}}, \mathbf{X}_\mathrm{i}) + n^{-\delta} < \tau < 1\}.$$

3. If τ is in B_n, estimate the quantile coefficients $\widehat{\boldsymbol{\theta}}^{\mathrm{Q}}$ at the nominal quantile level $\Gamma(1 - \pi(\widehat{\boldsymbol{\theta}}^{\mathrm{L}}, \mathbf{X}_\mathrm{i}) + n^{-\delta}; \mathbf{X}_\mathrm{i}, \widehat{\boldsymbol{\theta}}^{\mathrm{L}})$ and perform an interpolation between the quantile estimate and 1, which is the natural lower bound for microbial read count, at the change point $1 - \pi(\widehat{\boldsymbol{\theta}}^{\mathrm{L}}, \mathbf{X}_\mathrm{i})$. Pick the interpolated value at τ. If τ is in C_n, estimate the $\widehat{\boldsymbol{\theta}}^{\mathrm{Q}}$ at $\Gamma(\tau; \mathbf{X}_\mathrm{i}, \widehat{\boldsymbol{\theta}}^{\mathrm{L}})$ directly. If linear interpolation is used, the estimation of $\hat{Q}$ is shown as Supp. Fig. 1, and mathematically is

$$\hat{Q} = 0 \cdot \mathrm{I}(\tau \in A_n)$$
$$+ \left[\{\mathbf{X}_\mathrm{i}^\mathrm{T}\widehat{\boldsymbol{\theta}}^\mathrm{Q} \circ \Gamma\big(1 - \pi\big(\widehat{\boldsymbol{\theta}}^\mathrm{L}, \mathbf{X}_\mathrm{i}\big) + n^{-\delta}; \mathbf{X}_\mathrm{i}, \widehat{\boldsymbol{\theta}}^\mathrm{L}\big) - 1\} \cdot \frac{\tau - \{1 - \pi\big(\widehat{\boldsymbol{\theta}}^\mathrm{L}, \mathbf{X}_\mathrm{i}\big)\}}{n^{-\delta}} \right] \cdot \mathrm{I}(\tau \in B_n)$$
$$+ \left[\mathbf{X}_\mathrm{i}^\mathrm{T}\widehat{\boldsymbol{\theta}}^\mathrm{Q} \circ \Gamma\big(\tau; \mathbf{X}_\mathrm{i}, \widehat{\boldsymbol{\theta}}^\mathrm{L}\big) - 1\right] \cdot \mathrm{I}(\tau \in C_n) \qquad \text{(Supp. 2)}$$

Intuitively, the buffer zone B_n avoids the need to estimate the minimal quantiles of the non-zero part, preventing an explosive estimate around the zero-positive change point. The width of B_n, $n^{-\delta}$, is designed to converge more slowly than the logistic estimates, so the buffer will work and $\hat{Q}$ is bounded and reliable almost surely. The consistency of $\hat{Q}$ can be found elsewhere[2]. Other smooth methods can be used instead of the linear interpolation, to further control the estimate around the change point. There is a tradeoff when determining the width of B_n. A wider B_n controls the estimates well but the longer interpolation introduces more biases. While if B_n is too narrow, the estimates close to the change point might not be properly controlled. Practically, when the sample size is reasonably large, we use a value approaching $\frac{1}{2}$ as δ, such as 0.499. As a complement to Fig. 1b, the matching process on the piecewise estimated conditional quantile functions is depicted in Supp. Fig. 2.

However, when the sample size is severely limited, we might opt to omit the buffer zone B_n in estimation. A small sample size leads to a wide B_n, then the interpolation in B_n will induce substantial biases. In such extreme cases, reducing the biases is our primary goal, instead of controlling the explosive estimates that might occur.

Figures and tables

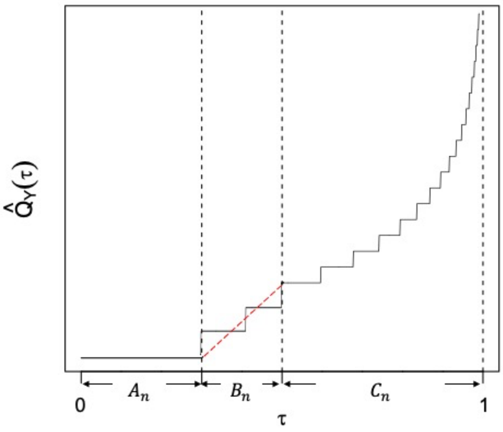

Supp. Fig. 1 | Piecewise estimation of conditional quantile function.

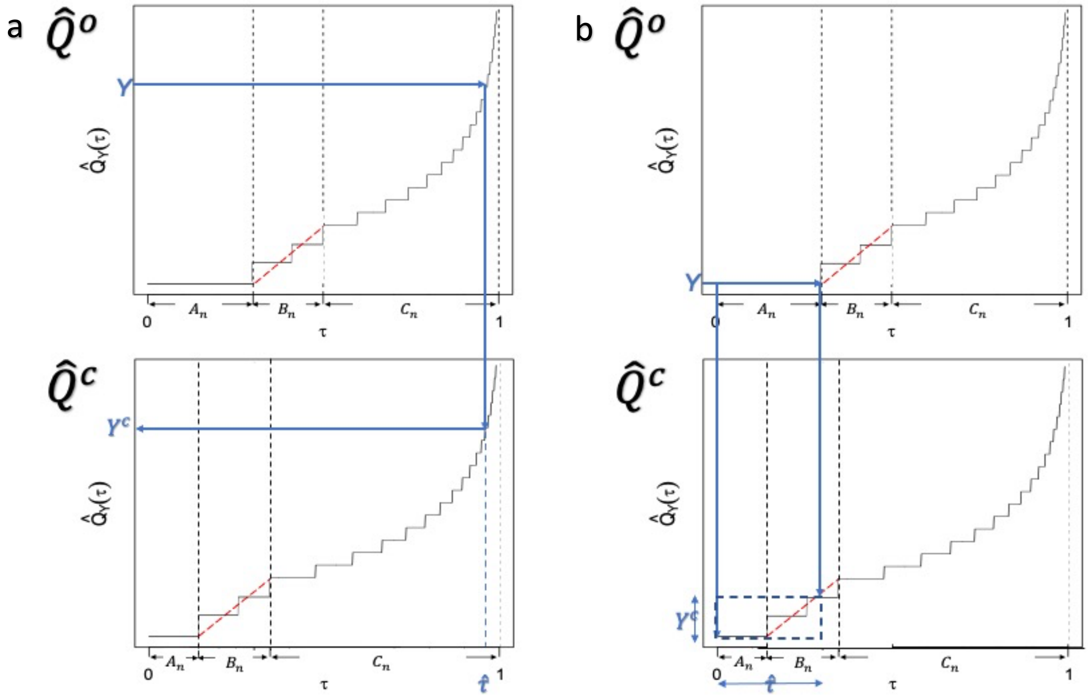

Supp. Fig. 2 | Correction with conditional quantile functions. a, Converting a non-zero count to the corrected non-zero count at the same quantile level. **b,** Converting a zero count to a non-zero count when the batch-free distribution is less sparse. Rounded average of all matched quantiles in the batch-free distribution is taken as the adjusted read count.

a

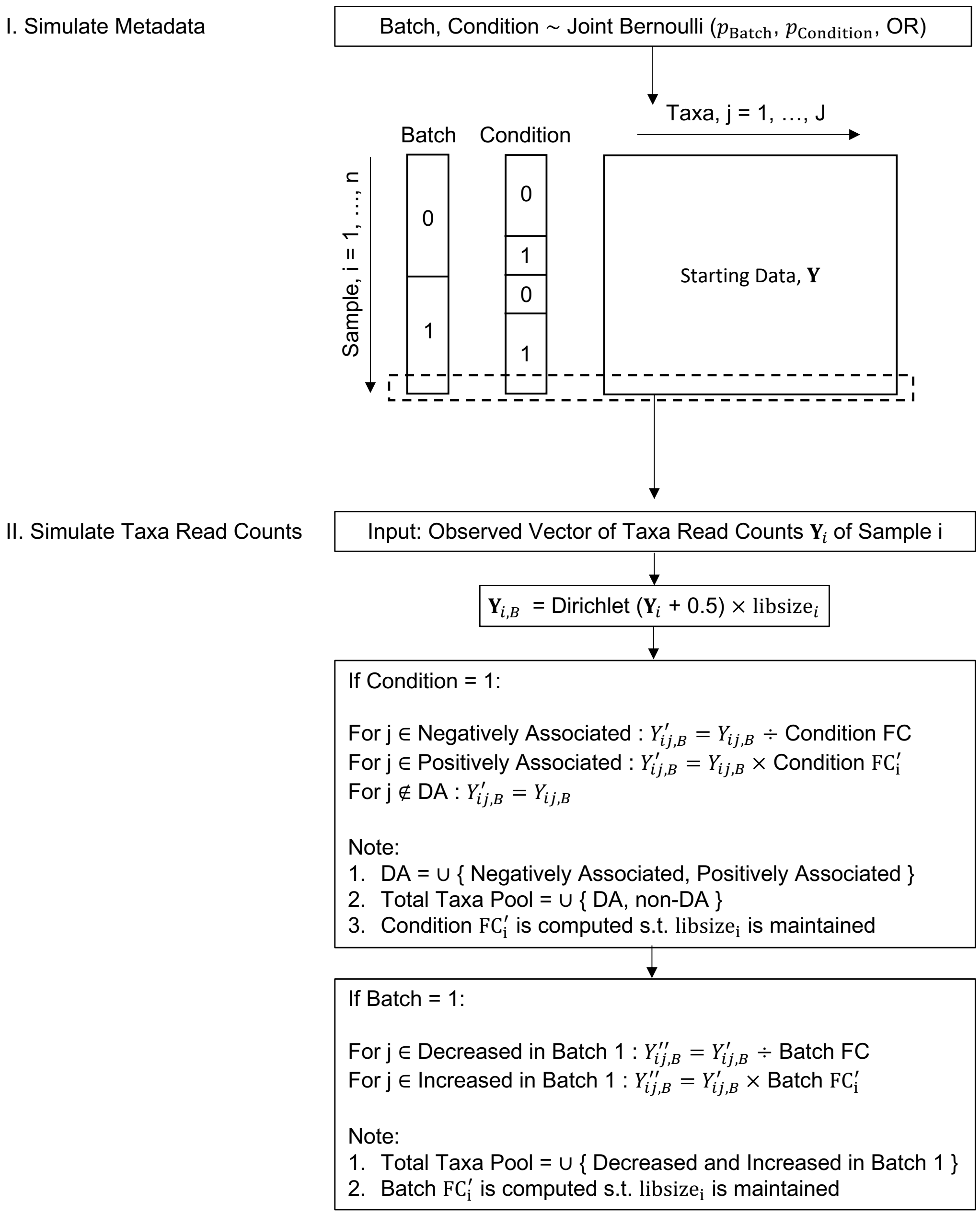

I. Simulate Metadata
Batch, Condition ~ Joint Bernoulli (p_{Batch}, $p_{\text{Condition}}$, OR)
Taxa, j = 1, …, J
Batch
Condition
Sample, i = 1, …, n
0
1
0
1
0
1
Starting Data, $\mathbf{Y}$
II. Simulate Taxa Read Counts
Input: Observed Vector of Taxa Read Counts $\mathbf{Y}_i$ of Sample i
$\mathbf{Y}_{i,B}$ = Dirichlet ($\mathbf{Y}_i$ + 0.5) × libsize_i
If Condition = 1:
For j ∈ Negatively Associated : $Y'_{ij,B} = Y_{ij,B}$ ÷ Condition FC
For j ∈ Positively Associated : $Y'_{ij,B} = Y_{ij,B}$ × Condition FC'_i
For j ∉ DA : $Y'_{ij,B} = Y_{ij,B}$
Note:
1. DA = ∪ { Negatively Associated, Positively Associated }
2. Total Taxa Pool = ∪ { DA, non-DA }
3. Condition FC'_i is computed s.t. libsize_i is maintained
If Batch = 1:
For j ∈ Decreased in Batch 1 : $Y''_{ij,B} = Y'_{ij,B}$ ÷ Batch FC
For j ∈ Increased in Batch 1 : $Y''_{ij,B} = Y'_{ij,B}$ × Batch FC'_i
Note:
1. Total Taxa Pool = ∪ { Decreased and Increased in Batch 1 }
2. Batch FC'_i is computed s.t. libsize_i is maintained

b

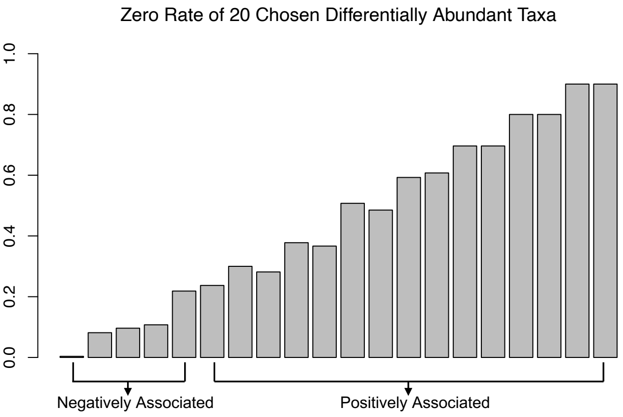

Zero Rate of 20 Chosen Differentially Abundant Taxa
0.0
0.2
0.4
0.6
0.8
1.0
Negatively Associated
Positively Associated

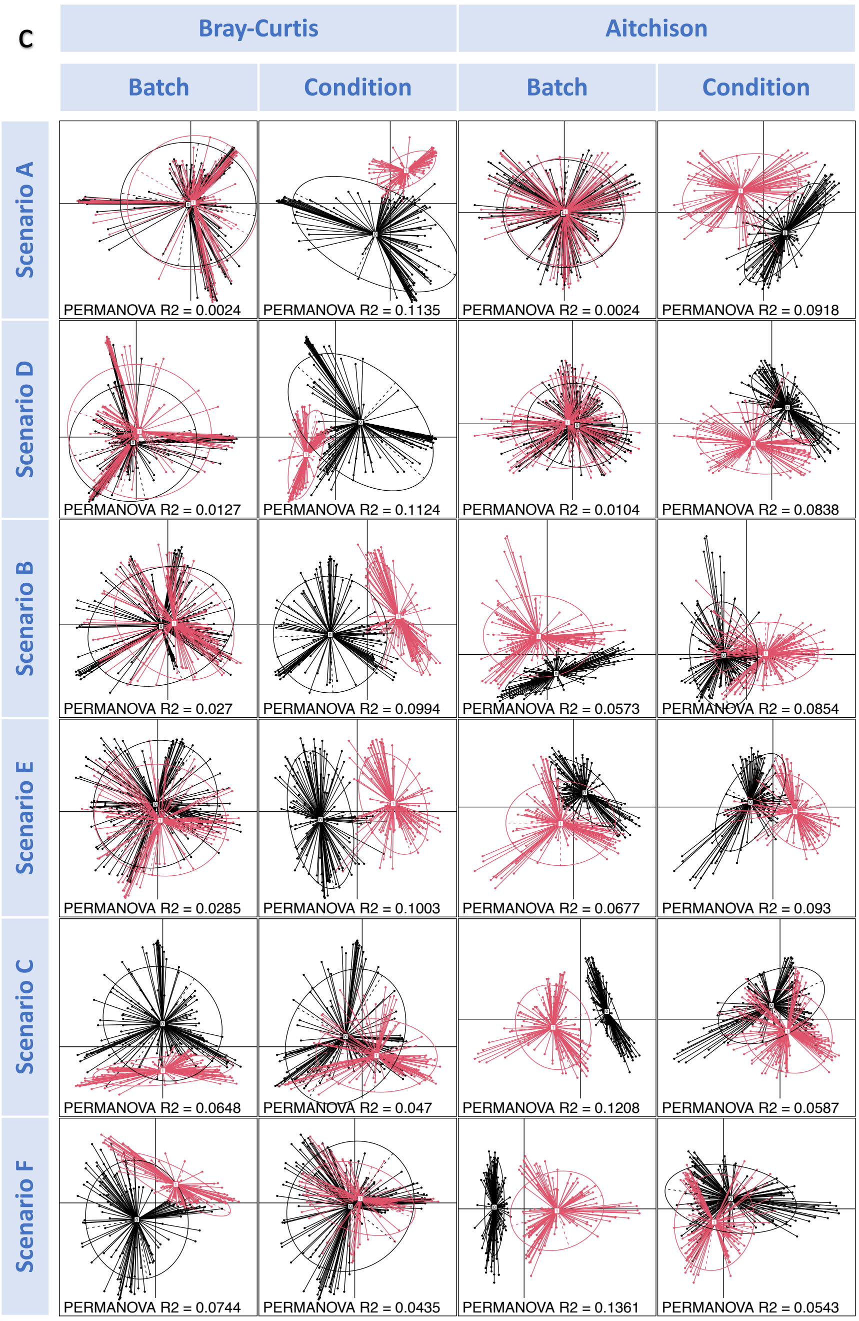

Supp. Fig. 3 | Simulation setting, workflow, and visualization of simulated data. a, Workflow of generating simulated data based on the starting data. Specification of fold change (FC) and odds ratio (OR) are provided in Results – Evaluation on simulated data. Rounded $Y''_{ij,B}$ is the simulated (bootstrapped) count of the j-th taxon in the i-th sample. The starting data only comprises the subset of MOMS-PI with body_site = vaginal and visit = 4, and with samples having less than 4000 library size and rare taxa presenting in less than 5% samples filtered out. **b,** Zero rates of the 20 chosen DA taxa in the starting data. The most abundant 5 taxa in the 20 are set to be negatively associated with Condition, because if they are set to be positively associated, their multiplied counts will be too large to maintain

libsize. **c,** PCoA plots of one simulated data for each of the six scenarios. In either Bray-Curtis or Aitchison dissimilarly, the visualization and PERMANOVA R^2 clearly show the three patterns, Null (Scenario A), Condition Effect > Batch Effect (Scenario B), and Condition Effect < Batch Effect (Scenario C). Scenario D, E, and F show similar patterns, respectively, with increased Batch Effect incorporating library size variability.

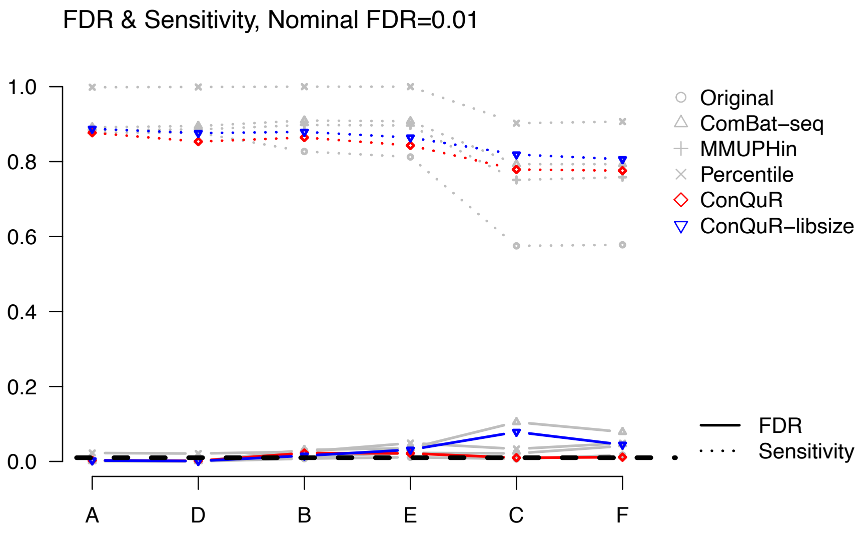

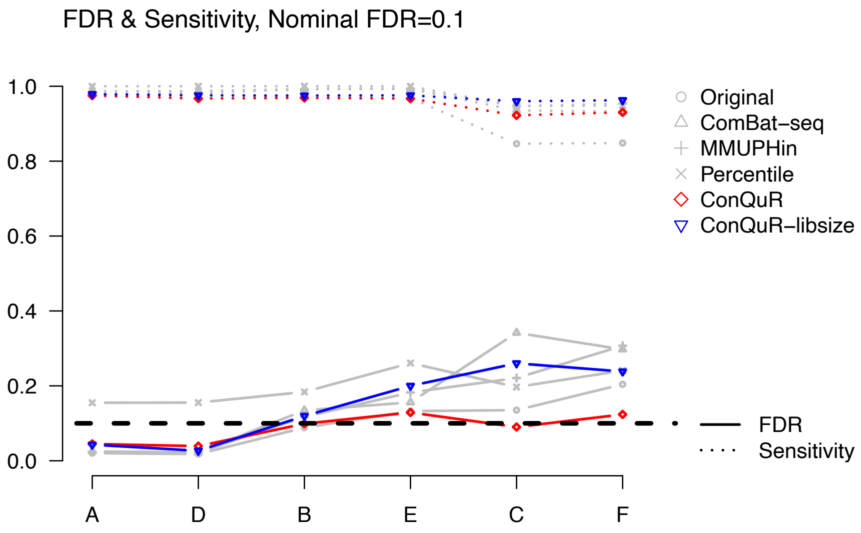

Supp. Fig. 4 | False discovery rate (FDR) and sensitivity of association analysis between taxa relative abundance and Condition, with additional nominal FDR cutoffs. In the plots, the scenarios are arranged on the x-axis with the order A, D, B, E, C, F because the two

Nulls are allocated together, followed by Condition Effect > Batch Effect, and then Condition Effect < Batch Effect. Color and the name of the corresponding method are shown on the right within the graph. The solid line is FDR and the dashed line is sensitivity. Approaches with FDR attained around the nominal level 0.01 or 0.1 are valid, then among the valid approaches, the higher sensitivity is preferred.

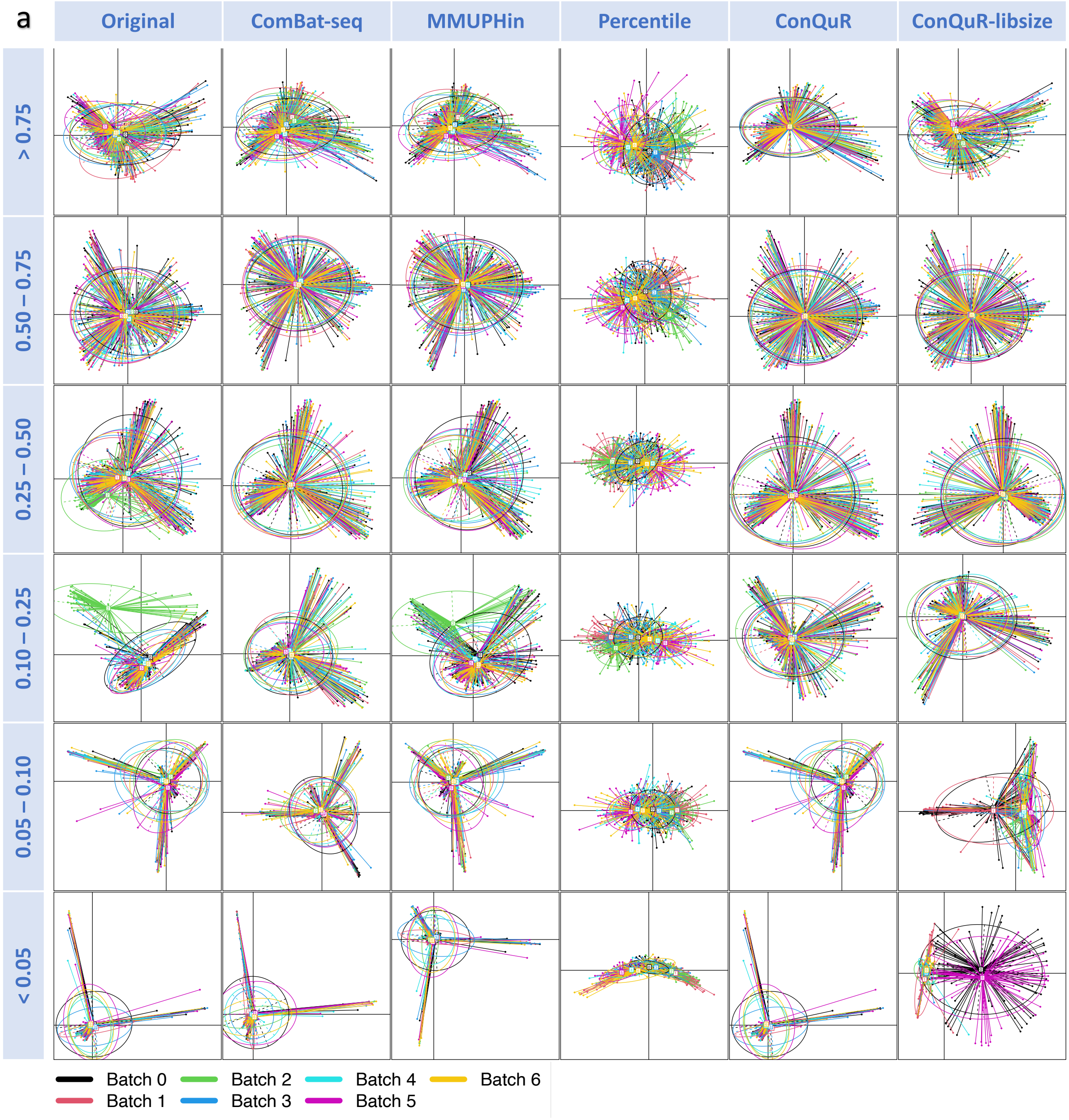

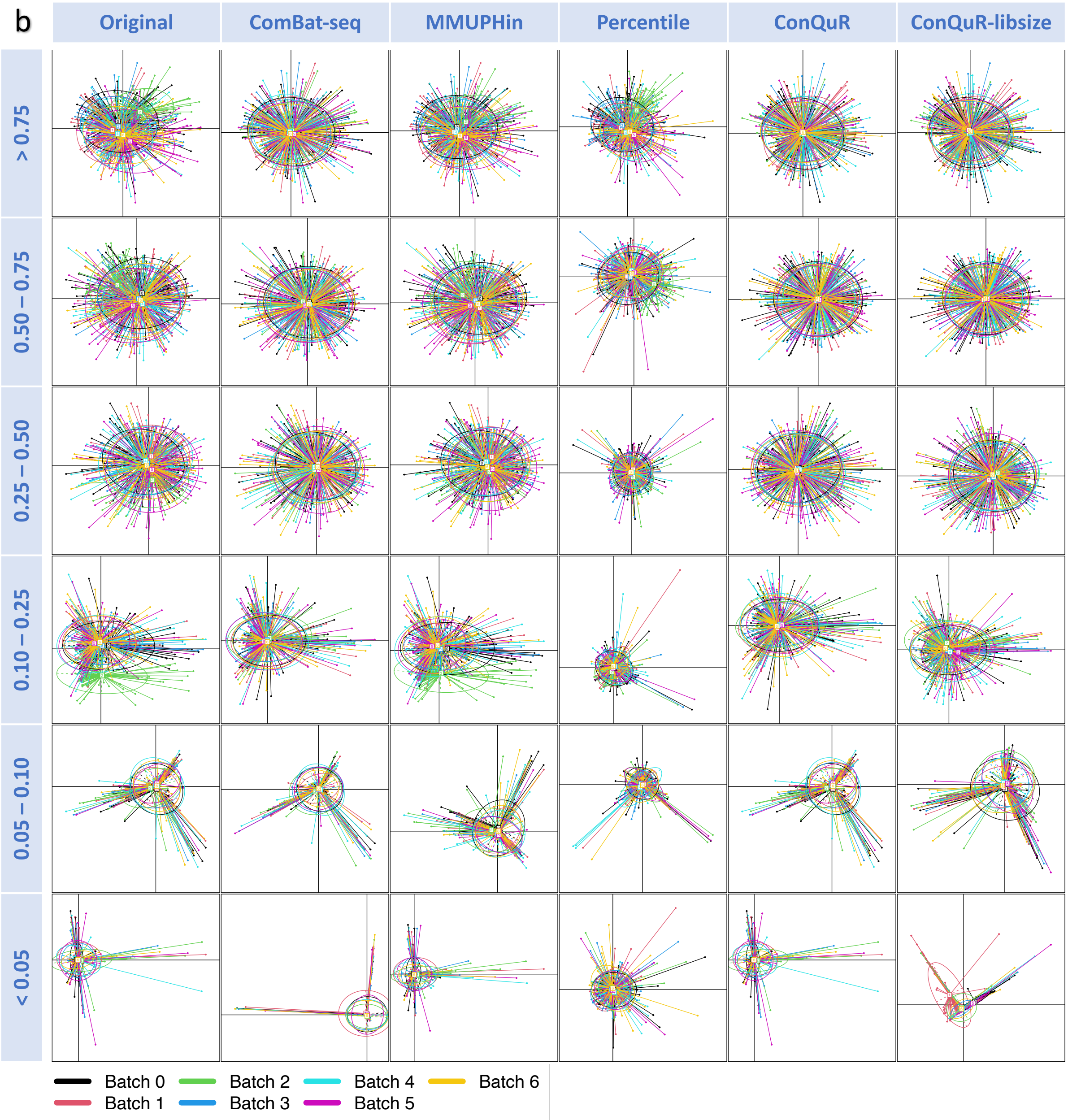

b
Original
ComBat-seq
MMUPHin
Percentile
ConQuR
ConQuR-libsize
> 0.75
0.50 – 0.75
0.25 – 0.50
0.10 – 0.25
0.05 – 0.10
< 0.05
Batch 0
Batch 1
Batch 2
Batch 3
Batch 4
Batch 5
Batch 6

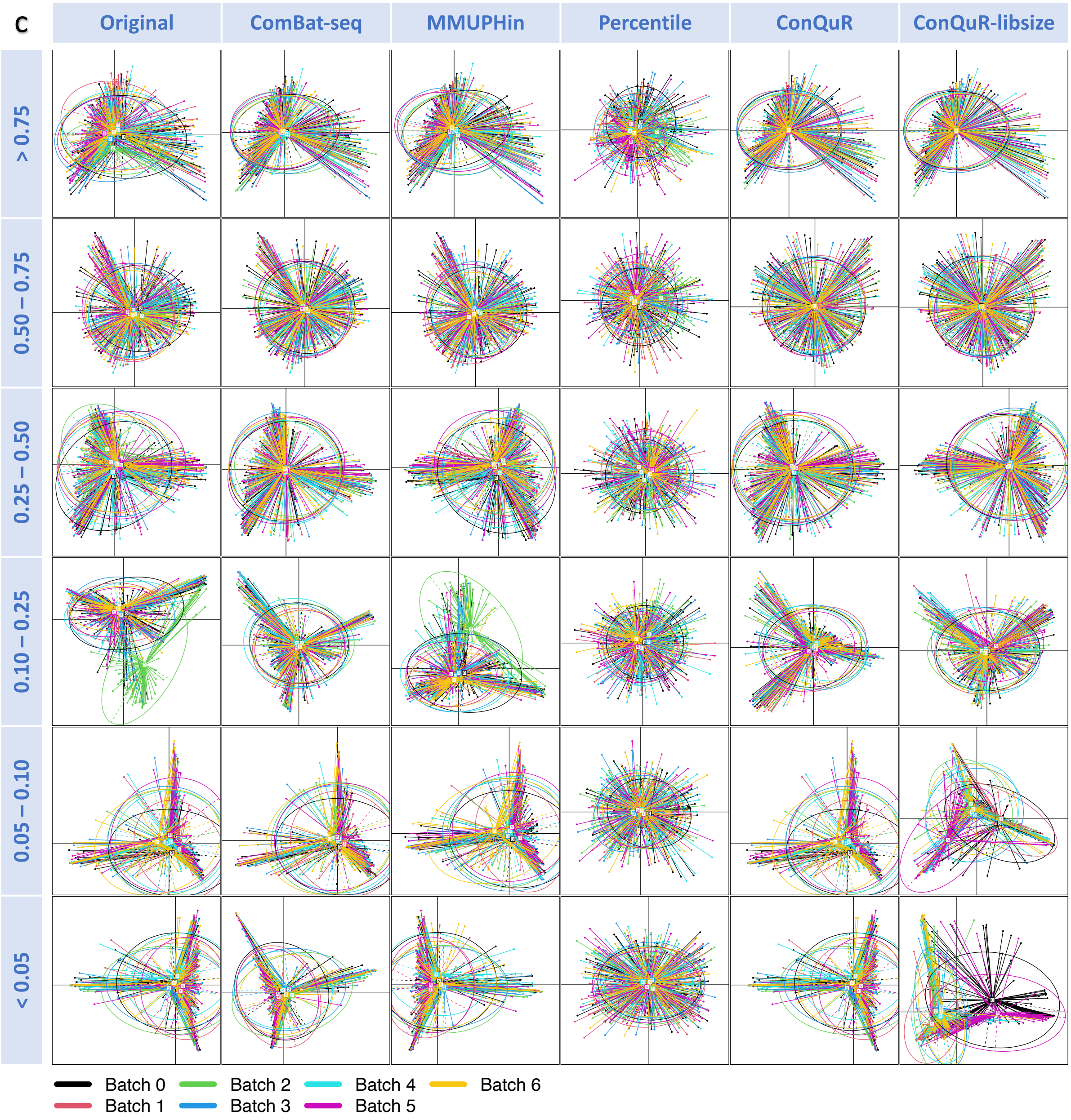

Supp. Fig. 5 | PCoA plots of CARDIA data clustered by batch ID on taxa with different prevalence. Corresponding colors of the batches are shown at the bottom within the graph. **a,** by Bray-Curtis dissimilarity on raw counts of taxa with prevalence >0.75, 0.5-0.75, 0.25-0.5, 0.1-0.25, 0.05-0.1, <0.05. Each point represents a sample with non-zero abundance for the taxa (note that fewer samples will have non-zero abundance for rarer taxa). Each ellipse represents a batch with the centroid indicating the mean. As an ellipse connects the 95% percentile of points for each batch, the size of the ellipse indicates the dispersion, and the angle indicates higher-order features of the batch. The more alignment of the ellipses is preferred. **b,** by Aitchison dissimilarity on the corresponding relative abundance of taxa with prevalence >0.75, 0.5-0.75, 0.25-0.5, 0.1-0.25, 0.05-0.1, <0.05. **c,** by GUniFrac dissimilarity

on the corresponding relative abundance of taxa with prevalence >0.75, 0.5-0.75, 0.25-0.5, 0.1-0.25, 0.05-0.1, <0.05.

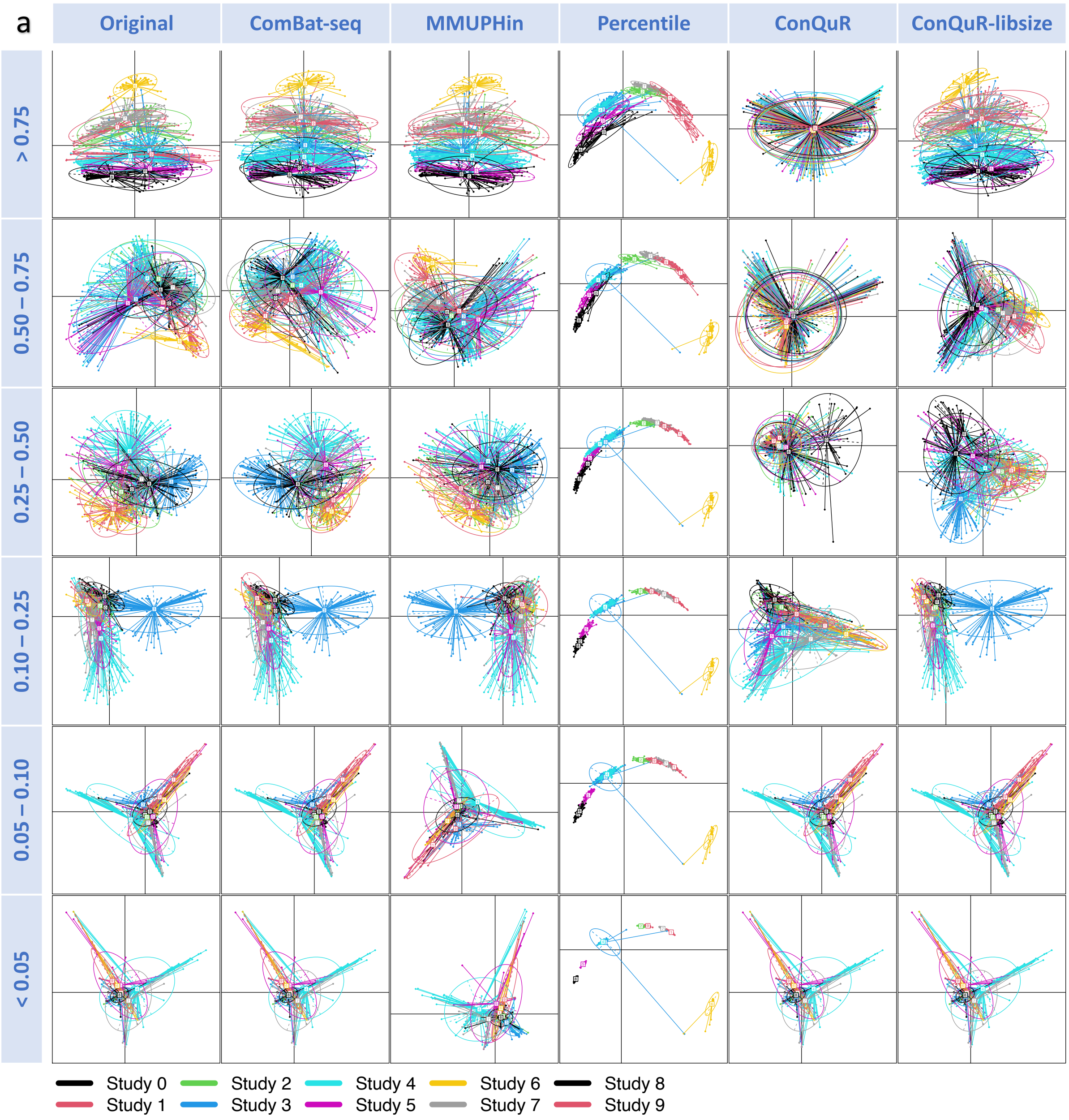

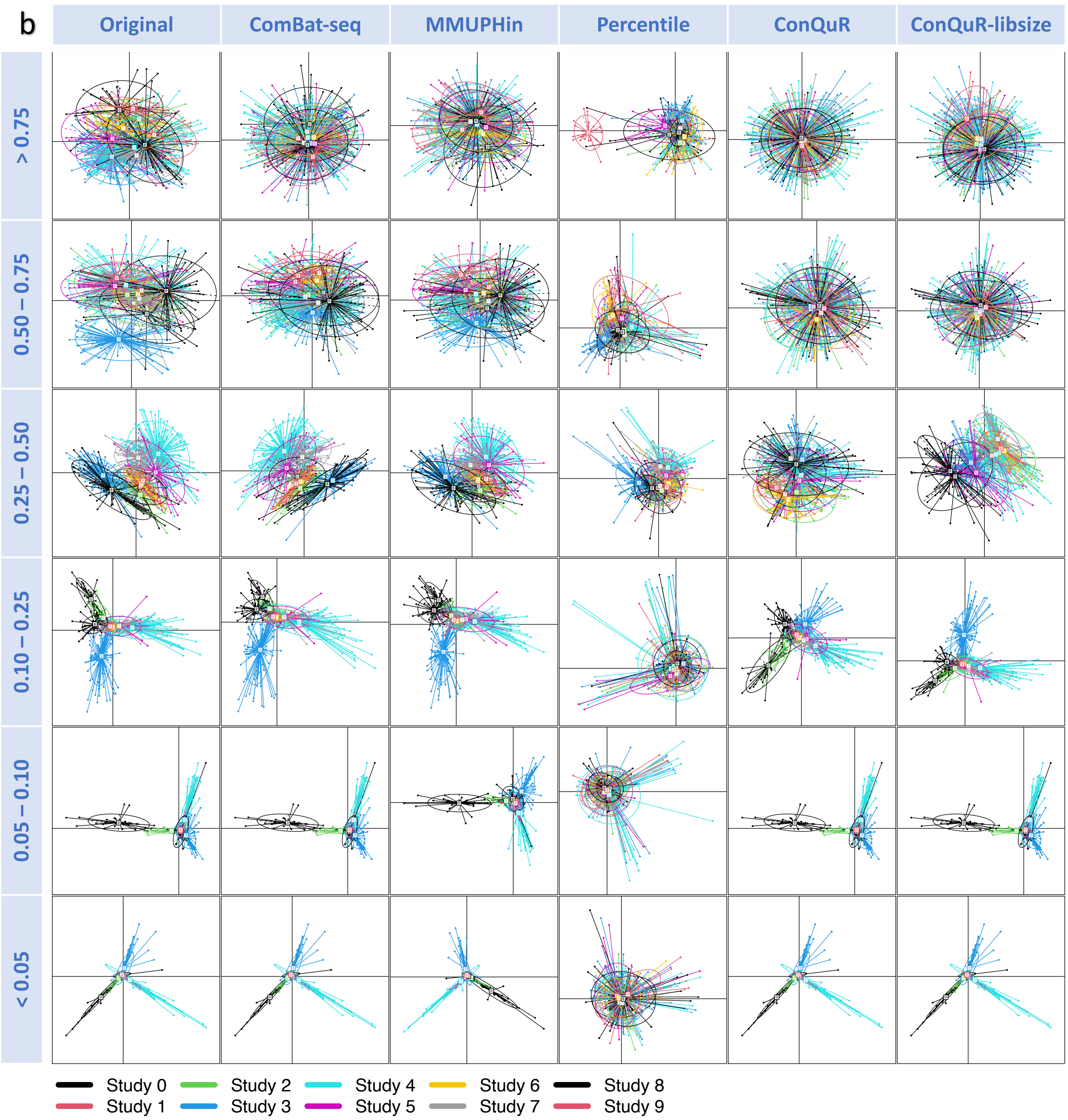

Supp. Fig. 6 | PCoA plots of HIVRC data clustered by study ID on taxa with different prevalence. Corresponding colors of the studies are shown at the bottom within the graph. **a,** by Bray-Curtis dissimilarity on raw counts of taxa with prevalence >0.75, 0.5-0.75, 0.25-0.5, 0.1-0.25, 0.05-0.1, <0.05. Each point represents a sample with non-zero abundance for the taxa (note that fewer samples will have non-zero abundance for rarer taxa). Each ellipse represents a batch with the centroid indicating the mean. As an ellipse connects the 95% percentile of points for each batch, the size of the ellipse indicates the dispersion, and the angle indicates higher-order features of the batch. The more alignment of the ellipses is preferred. **b,** by Aitchison dissimilarity on the corresponding relative abundance of taxa with prevalence >0.75, 0.5-0.75, 0.25-0.5, 0.1-0.25, 0.05-0.1, <0.05.

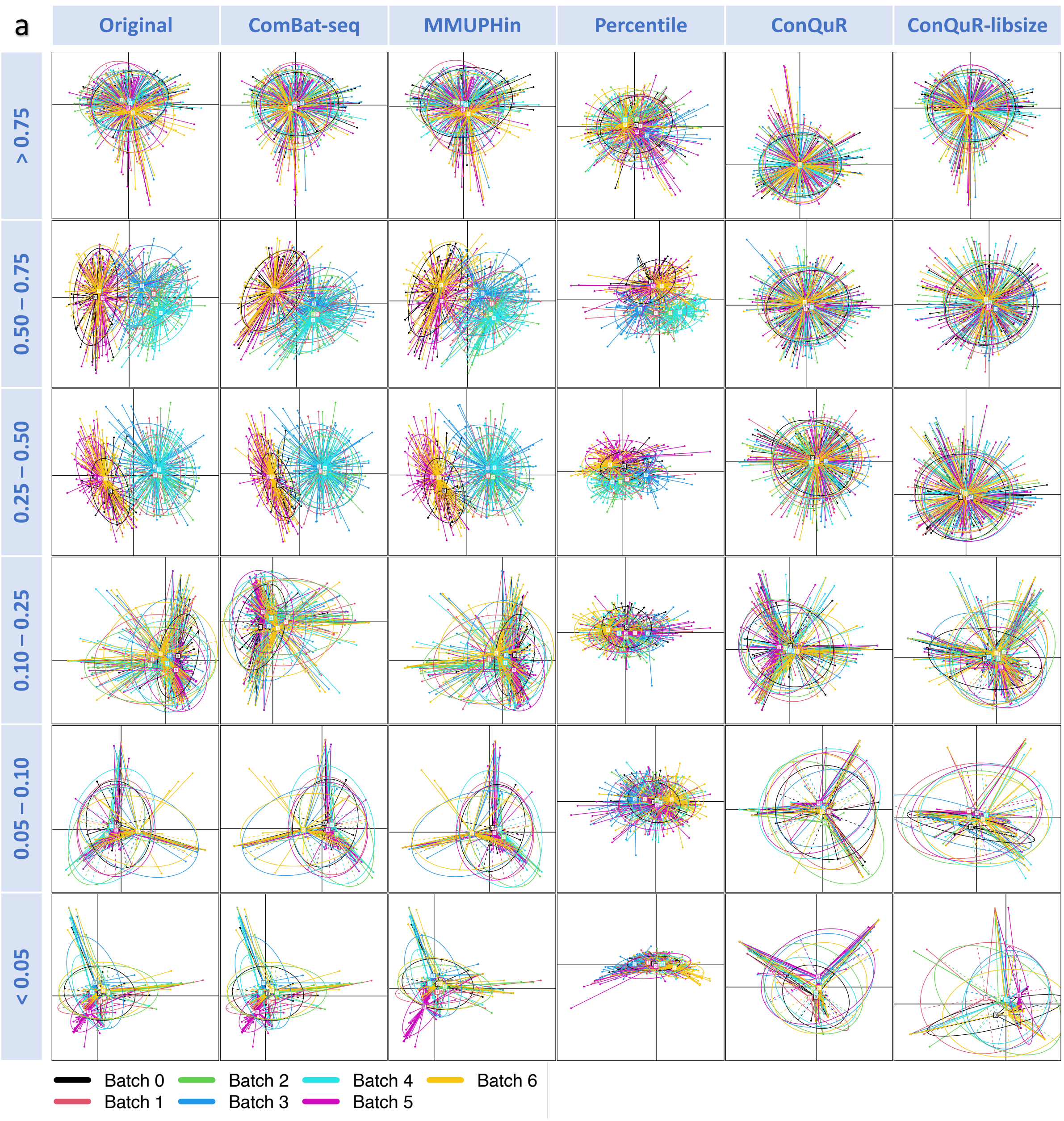
a
Original
ComBat-seq
MMUPHin
Percentile
ConQuR
ConQuR-libsize
> 0.75
0.50 – 0.75
0.25 – 0.50
0.10 – 0.25
0.05 – 0.10
< 0.05
Batch 0
Batch 1
Batch 2
Batch 3
Batch 4
Batch 5
Batch 6

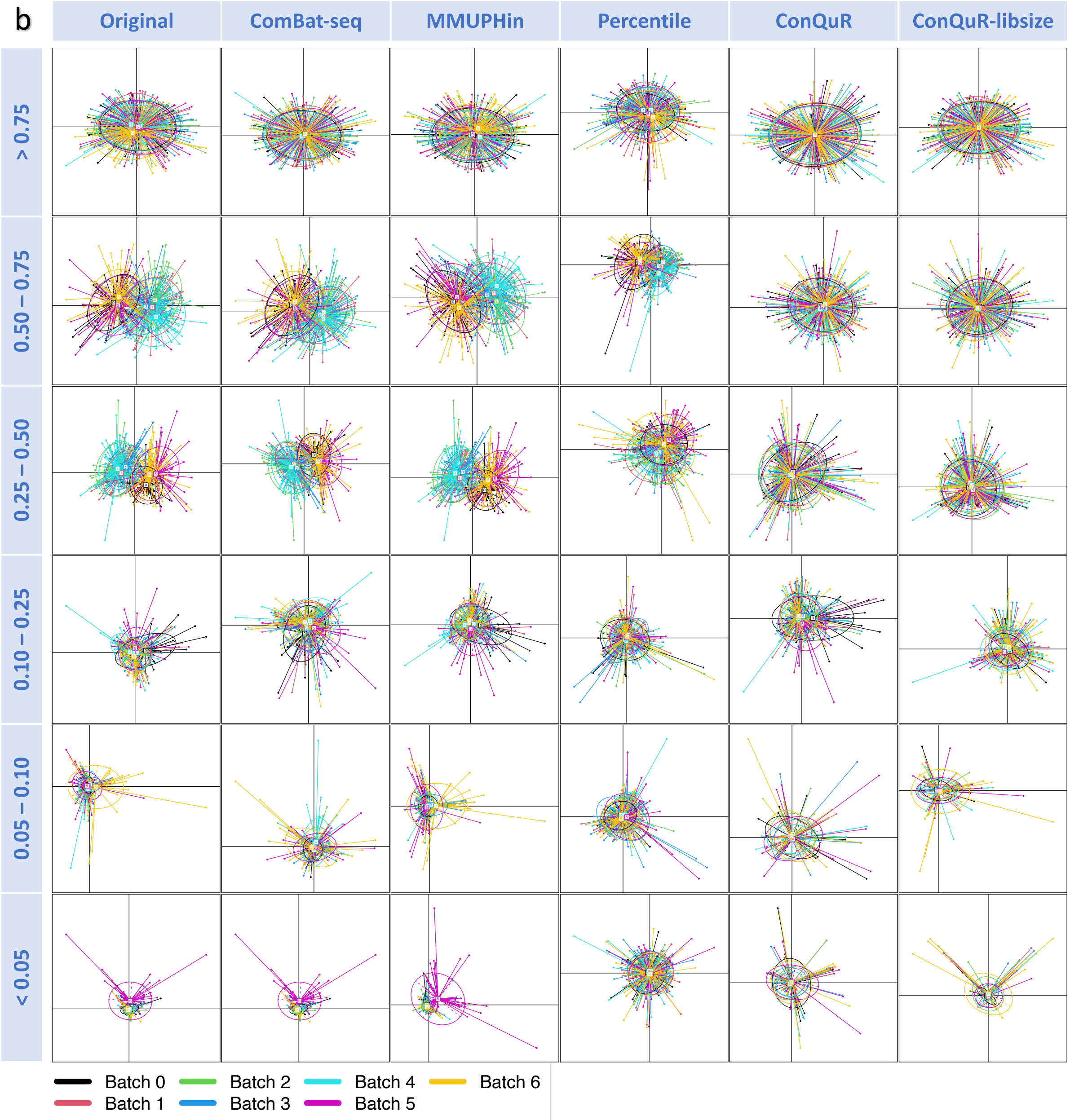
b
Original
ComBat-seq
MMUPHin
Percentile
ConQuR
ConQuR-libsize
> 0.75
0.50 – 0.75
0.25 – 0.50
0.10 – 0.25
0.05 – 0.10
< 0.05
Batch 0
Batch 1
Batch 2
Batch 3
Batch 4
Batch 5
Batch 6

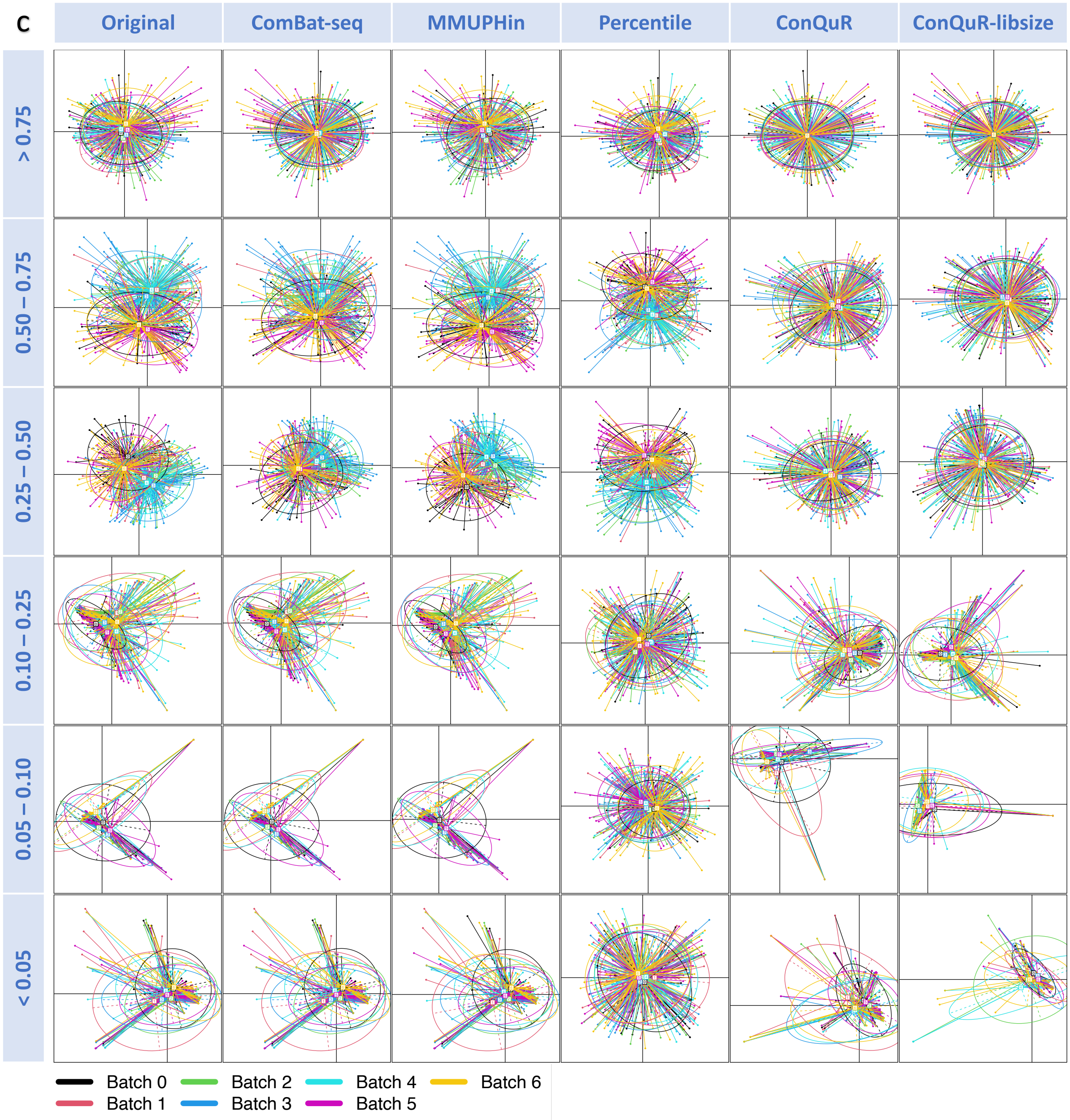

Supp. Fig. 7 | PCoA plots of MOUTH data clustered by batch ID on taxa with different prevalence. Corresponding colors of the batches are shown at the bottom within the graph. **a,** by Bray-Curtis dissimilarity on raw counts of taxa with prevalence >0.75, 0.5-0.75, 0.25-0.5, 0.1-0.25, 0.05-0.1, <0.05. Each point represents a sample with non-zero abundance for the taxa (note that fewer samples will have non-zero abundance for rarer taxa). Each ellipse represents a batch with the centroid indicating the mean. As an ellipse connects the 95% percentile of points for each batch, the size of the ellipse indicates the dispersion, and the angle indicates higher-order features of the batch. The more alignment of the ellipses is preferred. **b,** by Aitchison dissimilarity on the corresponding relative abundance of taxa with prevalence >0.75, 0.5-0.75, 0.25-0.5, 0.1-0.25, 0.05-0.1, <0.05. **c,** by GUniFrac dissimilarity

on the corresponding relative abundance of taxa with prevalence >0.75, 0.5-0.75, 0.25-0.5, 0.1-0.25, 0.05-0.1, <0.05.

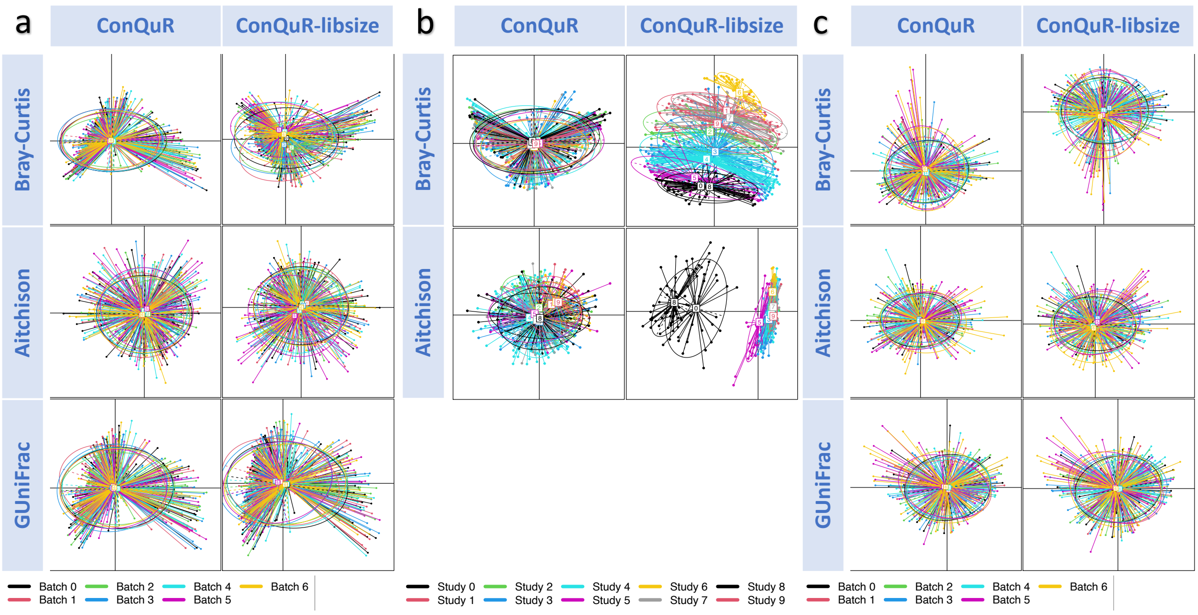

Supp. Fig. 8 | PCoA plots of corrected data by standard ConQuR and ConQuR-libsize without fine-tuning. Corresponding colors of the batches/studies are shown at the bottom within the graph. **a,** CARDIA data, by Bray-Curtis dissimilarity on raw counts, Aitchison and GUniFrac dissimilarities on the corresponding relative abundance. Each point represents a sample, and each ellipse represents a batch with the centroid indicating the mean. As an ellipse connects the 95% percentile of points for each batch, the size of the ellipse indicates the dispersion, and the angle indicates higher-order features of the batch. The more alignment of the ellipses is preferred. **b,** HIVRC data, by Bray-Curtis dissimilarity on raw counts, Aitchison dissimilarity on the corresponding relative abundance. **c,** MOUTH data, by Bray-Curtis dissimilarity on raw counts, Aitchison and GUniFrac dissimilarities on the corresponding relative abundance.

Supp. Tab. 1 | Summary of the 7 runs' microbial profiles and metadata of the CARDIA dataset.

Batch ID	Sample size	Library size	# Genera	SBP (mean (SD))	Gender = 1 (%)	Race = 1 (%)
0	96	67716-139803	258	118.00 (16.99)	48 (50.0)	60 (62.5)
1	89	53287-117222	241	117.82 (13.59)	52 (58.4)	61 (68.5)
2	90	61703-112177	237	121.11 (15.70)	47 (52.2)	44 (48.9)
3	82	69760-124721	262	119.21 (14.37)	42 (51.2)	47 (57.3)
4	94	84358-193182	259	119.55 (17.21)	48 (51.1)	46 (48.9)
5	94	90841-224737	280	121.35 (17.02)	56 (59.6)	46 (48.9)
6	88	71850-253856	277	117.94 (17.17)	57 (64.8)	45 (51.1)
			Shared: 183	p-value=0.585	p-value=0.329	p-value=0.034

Note: One-way ANOVA test and Pearson's Chi-squared test were used to detect whether there are significant differences in continuous (SBP) and categorial (Gender, Race) metadata, respectively, among the 7 runs.

Supp. Tab. 2 | Summary of the 10 sub-studies' microbial profiles and metadata of the HIVRC dataset.

Study ID	Author	Sample size	Library size	# Genera	HIV status = 1 (%)	Age (mean (SD))	Gender = 1 (%)	BMI (mean (SD))
0	Dillon[3]	31	20000	235	18 (58.1)	35.94 (10.63)	10 (32.3)	25.18 (4.44)
1	Dinh[4]	36	3500	157	21 (58.3)	47.66 (7.80)	8 (22.2)	25.45 (3.15)
2	Lozupone[5]	37	5500	262	24 (64.9)	36.21 (10.72)	8 (21.6)	25.61 (4.84)
3	Monaco[6]	110	194-10000	280	73 (66.4)	39.57 (9.77)	63 (57.3)	24.33 (4.48)
4	Noguera-Julian[7]	137	10000	295	122 (89.1)	43.28 (10.12)	30 (21.9)	24.15 (3.02)
5	Pinto-Cardoso[8]	42	15500	225	33 (78.6)	40.19 (10.14)	8 (19.0)	24.29 (4.09)
6	Serrano-Villar[9]	43	185-1000	143	34 (79.1)	41.79 (10.55)	7 (16.3)	24.32 (3.11)
7	Vesterbacka[10]	62	4000	268	47 (75.8)	46.94 (9.61)	31 (50.0)	26.45 (4.32)
8	Villanueva-Millan[11]	50	20000	351	30 (60.0)	47.38 (9.49)	17 (34.0)	25.73 (5.12)
9	Villar-Garcia[12]	24	5000	148	24 (100.0)	45.92 (9.34)	3 (12.5)	23.58 (3.74)
				Shared: 65	p-value<0.001	p-value<0.001	p-value<0.001	p-value=0.004

Note: One-way ANOVA test and Pearson's Chi-squared test were used to detect whether there are significant differences in continuous (Age, BMI) and categorial (HIV status, Gender) metadata, respectively, among the 10 sub-studies.

Supp. Tab. 3 | Summary of the 7 runs' microbial profiles and metadata of the MOUTH dataset.

Batch ID	Sample size	Library size	# Genera	HPV status	Race			Sexual orientation		
				= 1 (%)	= 0 (%)	1 (%)	2 (%)	= 0 (%)	1 (%)	2 (%)
0	42	6721-15931	156	6 (14.3)	34 (81.0)	5 (11.9)	3 (7.1)	36 (85.7)	3 (7.1)	3 (7.1)
1	46	5340-14342	141	10 (21.7)	38 (82.6)	6 (13.0)	2 (4.3)	41 (89.1)	1 (2.2)	4 (8.7)
2	56	7267-16694	150	8 (14.3)	41 (73.2)	10 (17.9)	5 (8.9)	49 (87.5)	3 (5.4)	4 (7.1)
3	77	5418-13520	152	6 (7.8)	63 (81.8)	9 (11.7)	5 (6.5)	71 (92.2)	4 (5.2)	2 (2.6)
4	89	7232-18607	179	9 (10.1)	76 (85.4)	9 (10.1)	4 (4.5)	83 (93.3)	3 (3.4)	3 (3.4)
5	89	2670-18610	187	14 (15.7)	50 (56.2)	28 (31.5)	11 (12.4)	68 (76.4)	8 (9.0)	13 (14.6)
6	87	6534-21761	174	13 (14.9)	64 (73.6)	20 (23.0)	3 (3.4)	74 (85.1)	8 (9.2)	5 (5.7)
			Shared: 112	p-value=0.396	p-value=0.003			p-value=0.104		

Note: One-way ANOVA test and Pearson's Chi-squared test were used to detect whether there are significant differences in the categorial (HPV status, Race, Sexual orientation, Smoking status) metadata among the 7 runs.

Supp. Tab. 3 (cont.) | Summary of the 7 runs' microbial profiles and metadata of the MOUTH dataset.

Batch ID	Sample size	Smoking status		
		= 0 (%)	1 (%)	2 (%)
0	42	24 (57.1)	14 (33.3)	4 (9.5)
1	46	29 (63.0)	12 (26.1)	5 (10.9)
2	56	31 (55.4)	20 (35.7)	5 (8.9)
3	77	46 (59.7)	25 (32.5)	6 (7.8)
4	89	55 (61.8)	25 (28.1)	9 (10.1)
5	89	50 (56.2)	23 (25.8)	16 (18.0)
6	87	45 (51.7)	27 (31.0)	15 (17.2)
		p-value=0.661		

Note: One-way ANOVA test and Pearson's Chi-squared test were used to detect whether there are significant differences in the categorial (HPV status, Race, Sexual orientation, Smoking status) metadata among the 7 runs.

Supp. Tab. 4 | Fitting strategies selected for taxa with different prevalence by fine-tuned ConQuR and ConQuR-libsize on the CARDIA data.

	0 – 0.1	0.1 – 0.2	0.2 – 0.3	0.3 – 0.4	0.4 – 0.5	0.5 – 0.6	0.6 – 0.7	0.7 – 0.8	0.8 – 0.9	0.9 - 1
Original	X									
QQ								X	X	X
Logistic Lasso		X	X	X	X					
Quantile type		Lasso	Lasso	Lasso	Lasso	Lasso	Lasso			
Lambda		2p/n	2p/n	2p/n	2p/n	2p/n	2p/n			
Interpolation					X	X	X			

Note: To interpret the model, e.g., for taxa with prevalence < 0.1 in CARDIA, the optimal choice is to keep the original data; for taxa with 0.1 < prevalence < 0.2, ConQuR / ConQuR-libsize with logistic-LASSO, quantile-LASSO (with $\lambda = \frac{2p}{n}$), without interpolation in the piece-wise estimation strategy is selected; for taxa with 0.5 < prevalence < 0.6, ConQuR / ConQuR-libsize with standard logistic, quantile-LASSO (with $\lambda = \frac{2p}{n}$), with interpolation in the piece-wise estimation strategy is selected; for taxa with 0.7 < prevalence < 0.8, ConQuR / ConQuR-libsize with simple quantile-quantile matching is selected.

Supp. Tab. 5 | Fitting strategies selected for taxa with different prevalence by fine-tuned ConQuR and ConQuR-libsize on the HIVRC data.

	0 – 0.1	0.1 – 0.2	0.2 – 0.3	0.3 – 0.4	0.4 – 0.5	0.5 – 0.6	0.6 – 0.7	0.7 – 0.8	0.8 – 0.9	0.9 - 1
Original	X									
QQ										X
Logistic Lasso		X	X	X	X		X	X		
Quantile type		Lasso	composite	composite	Lasso	Lasso	Lasso	Lasso	Lasso	
Lambda		2p/log(n)	2p/log(n)	2p/n	2p/log(n)	2p/n	2p/log(n)	2p/n	2p/n	
Interpolation								X		

Note: To interpret the model, e.g., for taxa with prevalence < 0.1 in HIVRC, the optimal choice is to keep the original data; for taxa with 0.1 < prevalence < 0.2, ConQuR / ConQuR-libsize with logistic-LASSO, quantile-LASSO (with $\lambda = \frac{2p}{\log n}$), without interpolation in the piece-wise estimation strategy is selected; for taxa with 0.2 < prevalence < 0.3, ConQuR / ConQuR-libsize with logistic-LASSO, composite quantile (with $\lambda = \frac{2p}{\log n}$), without interpolation in the piece-wise estimation strategy is selected; similarly for the remaining intervals of prevalence.

Supp. Tab. 6 | Fitting strategies selected for taxa with different prevalence by fine-tuned ConQuR and ConQuR-libsize on the MOUTH data.

	0 – 0.1	0.1 – 0.2	0.2 – 0.3	0.3 – 0.4	0.4 – 0.5	0.5 – 0.6	0.6 – 0.7	0.7 – 0.8	0.8 – 0.9	0.9 - 1
Original										
QQ									X	X
Logistic Lasso		X	X				X			
Quantile type	Lasso	Lasso	Lasso	Lasso	Lasso	Lasso	Lasso	Lasso		
Lambda	2p/n	2p/log(n)	2p/n	2p/log(n)	2p/n	2p/log(n)	2p/log(n)	2p/n		
Interpolation			X			X	X	X		

Note: To interpret the model, e.g., for taxa with prevalence < 0.1 in MOUTH, ConQuR / ConQuR-libsize with standard logistic, quantile-LASSO (with $\lambda = \frac{2p}{n}$), without interpolation in the piece-wise estimation strategy is selected; for taxa with 0.1 < prevalence < 0.2, ConQuR / ConQuR-libsize with logistic-LASSO, quantile-LASSO (with $\lambda = \frac{2p}{\log n}$), without interpolation in the piece-wise estimation strategy is selected; for taxa with 0.2 < prevalence < 0.3, ConQuR / ConQuR-libsize with logistic-LASSO, quantile-LASSO (with $\lambda = \frac{2p}{n}$), with interpolation in the piece-wise estimation strategy is selected; similarly for the remaining intervals of prevalence.

References

1. Machado, J.A.F. & Silva, J.S. Quantiles for counts. *Journal of the American Statistical Association* **100**, 1226-1237 (2005).
2. Ling, W., Cheng, B., Wei, Y., Willey, J.Z. & Cheung, Y.K. Statistical inference in quantile regression for zero-inflated outcomes. *Statistica Sinica* **32**, 1411-1433 (2022).
3. Dillon, S. et al. An altered intestinal mucosal microbiome in HIV-1 infection is associated with mucosal and systemic immune activation and endotoxemia. *Mucosal immunology* **7**, 983-994 (2014).
4. Dinh, D.M. et al. Intestinal microbiota, microbial translocation, and systemic inflammation in chronic HIV infection. *The Journal of infectious diseases* **211**, 19-27 (2015).
5. Lozupone, C.A. et al. Alterations in the gut microbiota associated with HIV-1 infection. *Cell host & microbe* **14**, 329-339 (2013).
6. Monaco, C.L. et al. Altered virome and bacterial microbiome in human immunodeficiency virus-associated acquired immunodeficiency syndrome. *Cell host & microbe* **19**, 311-322 (2016).
7. Noguera-Julian, M. et al. Gut microbiota linked to sexual preference and HIV infection. *EBioMedicine* **5**, 135-146 (2016).
8. Pinto-Cardoso, S. et al. Fecal bacterial communities in treated HIV infected individuals on two antiretroviral regimens. *Scientific reports* **7**, 1-10 (2017).
9. Serrano-Villar, S. et al. The effects of prebiotics on microbial dysbiosis, butyrate production and immunity in HIV-infected subjects. *Mucosal immunology* **10**, 1279-1293 (2017).
10. Vesterbacka, J. et al. (Nature Publishing Group, 2017).
11. Villanueva-Millán, M.J., Pérez-Matute, P., Recio-Fernández, E., Lezana Rosales, J.M. & Oteo, J.A. Differential effects of antiretrovirals on microbial translocation and gut microbiota composition of HIV-infected patients. *Journal of the International AIDS Society* **20**, 21526 (2017).
12. Villar-Garcia, J. et al. Impact of probiotic Saccharomyces boulardii on the gut microbiome composition in HIV-treated patients: a double-blind, randomised, placebo-controlled trial. *PloS one* **12**, e0173802 (2017).
