# Supplementary material for: Batch effects removal for microbiome data via conditional quantile regression
Source: Nat Commun. 2022 Sep 15;13:5418. doi: 10.1038/s41467-022-33071-9 (PMC9477887; doi:10.1038/s41467-022-33071-9)
Supplement: Supplementary file 3 — Reporting Summary [file 41467_2022_33071_MOESM3_ESM.pdf]

nature portfolio

Corresponding author(s): Michael C. Wu, Ni Zhao

Last updated by author(s): Aug 23, 2022

Reporting Summary

Nature Portfolio wishes to improve the reproducibility of the work that we publish. This form provides structure for consistency and transparency in reporting. For further information on Nature Portfolio policies, see our Editorial Policies and the Editorial Policy Checklist.

Statistics

For all statistical analyses, confirm that the following items are present in the figure legend, table legend, main text, or Methods section.

n/a	Confirmed	
☐	☒	The exact sample size (*n*) for each experimental group/condition, given as a discrete number and unit of measurement
☒	☐	A statement on whether measurements were taken from distinct samples or whether the same sample was measured repeatedly
☐	☒	The statistical test(s) used AND whether they are one- or two-sided<br>*Only common tests should be described solely by name; describe more complex techniques in the Methods section.*
☐	☒	A description of all covariates tested
☐	☒	A description of any assumptions or corrections, such as tests of normality and adjustment for multiple comparisons
☐	☒	A full description of the statistical parameters including central tendency (e.g. means) or other basic estimates (e.g. regression coefficient) AND variation (e.g. standard deviation) or associated estimates of uncertainty (e.g. confidence intervals)
☐	☒	For null hypothesis testing, the test statistic (e.g. *F*, *t*, *r*) with confidence intervals, effect sizes, degrees of freedom and *P* value noted<br>*Give P values as exact values whenever suitable.*
☒	☐	For Bayesian analysis, information on the choice of priors and Markov chain Monte Carlo settings
☒	☐	For hierarchical and complex designs, identification of the appropriate level for tests and full reporting of outcomes
☒	☐	Estimates of effect sizes (e.g. Cohen's *d*, Pearson's *r*), indicating how they were calculated

Our web collection on statistics for biologists contains articles on many of the points above.

Software and code

Policy information about availability of computer code

Data collection	The CARDIA data were processed through DADA2, v 1.18. The HIVRC data were processed through Resphera Insight v 2.2. The MOUTH data were processed through QIIME2, v 2021.4.
Data analysis	The R package ConQuR is available at https://github.com/wdl2459/ConQuR. A vignette demonstrating use of ConQuR, data analysis, visual and numerical results generation is included (can be accessed at https://wdl2459.github.io/ConQuR/ConQuR.Vignette.html).

For manuscripts utilizing custom algorithms or software that are central to the research but not yet described in published literature, software must be made available to editors and reviewers. We strongly encourage code deposition in a community repository (e.g. GitHub). See the Nature Portfolio guidelines for submitting code & software for further information.

Data

Policy information about availability of data

All manuscripts must include a data availability statement. This statement should provide the following information, where applicable:

- Accession codes, unique identifiers, or web links for publicly available datasets
- A description of any restrictions on data availability
- For clinical datasets or third party data, please ensure that the statement adheres to our policy

The MOM-PI data are provided in the Bioconductor R package, HMP2Data, v 1.10. The CARDIA data can only be shared upon request due to the CARDIA Study Publications Policy. A request can be made by submitting a CARDIA Data Set Request-Intent to Analyze Form to the CARDIA Coordinating Center, University of Alabama at Birmingham, at https://www.cardia.dopm.uab.edu/publications-2/publications-documents. The reference database, Silva, can be found at https://www.arb-silva.de/. The integrated HIVRC data are available in Synapse under accession code syn18406854, https://www.synapse.org/#!Synapse:syn18406854. The MOUTH data are available in Synapse under accession code syn26529406, https://www.synapse.org/#!Synapse:syn26529406.

Field-specific reporting

Please select the one below that is the best fit for your research. If you are not sure, read the appropriate sections before making your selection.

☒ Life sciences ☐ Behavioural & social sciences ☐ Ecological, evolutionary & environmental sciences

For a reference copy of the document with all sections, see nature.com/documents/nr-reporting-summary-flat.pdf

Life sciences study design

All studies must disclose on these points even when the disclosure is negative.

Sample size	Sample sizes of microbiome data were determined by the size of available CARDIA, HIV sub-studies, and MOUTH data.
Data exclusions	Microbial taxa with only zero lineages and samples with missing covariates (metadata) were excluded.
Replication	We repeated the same analysis procedure on random sub-sampling of the two data sets, all attempts were successful (e.g., the analysis in the vignette, https://wdl2459.github.io/ConQuR/ConQuR.Vignette.html).
Randomization	There was no randomization in the study. Randomization is not relevant to our work because it is primarily methodological modeling of existing and published data, and is not human subject. Also, important covariates of metadata were adjusted when we did batch removal analysis.
Blinding	There was no blinding in the study. Blinding is not relevant to to our work because it is primarily methodological modeling of existing and published data, and is not human subject.

Reporting for specific materials, systems and methods

We require information from authors about some types of materials, experimental systems and methods used in many studies. Here, indicate whether each material, system or method listed is relevant to your study. If you are not sure if a list item applies to your research, read the appropriate section before selecting a response.

Materials & experimental systems

n/a	Involved in the study	
☒	☐	Antibodies
☒	☐	Eukaryotic cell lines
☒	☐	Palaeontology and archaeology
☒	☐	Animals and other organisms
☒	☐	Human research participants
☒	☐	Clinical data
☒	☐	Dual use research of concern

Methods

n/a	Involved in the study	
☒	☐	ChIP-seq
☒	☐	Flow cytometry
☒	☐	MRI-based neuroimaging
